# Ameloblastic Fibro-Odontoma with an *FGFR1* Mutation: A Case Report

**DOI:** 10.1007/s12105-025-01758-2

**Published:** 2025-02-05

**Authors:** Oumaima Aouam, Nard G. Janssen, Wendy W. J. de Leng, Gerben E. Breimer

**Affiliations:** 1 Department of Pathology, University Medical Center Utrecht, Utrecht, 3584 CX The Netherlands; 2 Department of Oral and Maxillofacial Pediatric Surgery, University Medical Center Utrecht, Utrecht, The Netherlands

**Keywords:** Ameloblastic fibro-odontoma, Odontogenic tumors, Developing odontoma, Molecular pathology, FGFR mutation

## Abstract

**Purpose:**

Ameloblastic fibro-odontoma (AFO) is a rare benign mixed odontogenic tumor that, after being classified for years as a distinct entity, was redefined as a “developing odontoma” in the 2017 World Health Organization classification. This article presents a unique case of an AFO with an *FGFR1* mutation.

**Methods:**

We present a case of an 8-year-old child with a slowly progressive swelling in the lower left mandible. Next-generation sequencing (TSO500 panel) was performed.

**Results:**

Panoramic radiography revealed an odontogenic tumor; therefore, a transoral enucleation was performed. Pathological microscopic examination confirmed the diagnosis of AFO, and next-generation sequencing detected an *FGFR1* mutation.

**Conclusion:**

The presence of an *FGFR1* mutation in an AFO may suggest a closer biological relationship between ameloblastic fibroma and AFO, potentially distinguishing it from odontomas. Further research, including genetic studies, is needed to enhance our understanding and refine the classification of these tumors.

## Introduction

Ameloblastic fibro-odontomas (AFO), previously considered a distinct entity among rare odontogenic tumors, were reclassified in the 2017 World Health Organization classification as “developing odontomas [1].” They account for 1–3% of all odontogenic tumors [2], and are most commonly observed in children, with a mean age of 9.6 years, displaying a male predilection [3, 4]. Typically asymptomatic, these tumors are often associated with slow jaw growth or delayed tooth eruption and are most frequently found in the posterior region of the mandible [3, 4].

Radiologically, AFO typically presents as well-demarcated radiolucent lesions containing radiopaque areas, often associated with impacted teeth [3, 4]. This radiographic heterogeneity reflects the tumor’s mixed histological nature, including epithelial and mesenchymal components with dentine and enamel formation. It shares histological features with ameloblastic fibroma and complex odontoma.

The preferred treatment for AFO is conservative surgical removal due to its solid and well-demarcated nature [3, 4]. Depending on the clinical context, efforts may be made to preserve unerupted teeth. There is a risk of recurrence of AFO, and there are documented reports of AFO undergoing malignant transformation into ameloblastic fibrosarcoma [3, 4]. Hence, there is significance in recognizing and correctly diagnosing these tumors.

Currently, only limited information is available on the molecular biology of these tumors, making this case report an important contribution to understanding their molecular aspects. This report presents a case of AFO with a Fibroblast Growth Factor Receptor 1 (*FGFR1*) mutation, detailing the clinical presentation, radiographic features, and histological findings.

## Case

An 8-year-old child was referred to the department of maxillofacial surgery of the University Medical Centre Utrecht because of a slowly progressive swelling in the lower left mandible. Physical examination showed the absence of the lower left first permanent molar, no complaints of pain or hypesthesia, and a hard swelling in the left posterior buccal aspect of the mandible.

**Fig. 1:**
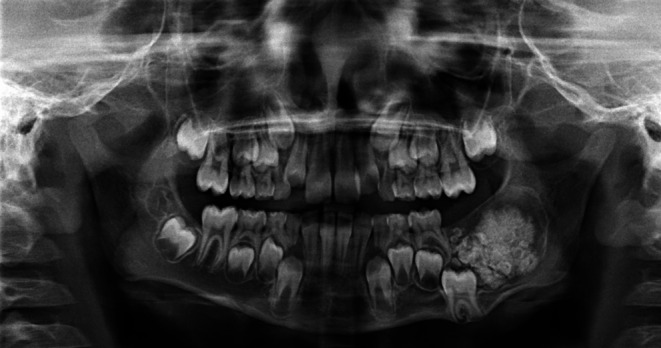
A panoramic radiograph obtained during the initial visit showed a circumscribed unilocular lesion in the lower left mandible containing partly amorphous and partly tooth-like radioopacities. Note the caudally displaced first permanent lower left molar and an absence of the tooth bud of the second lower left molar

A panoramic radiographic examination showed a circumscribed unilocular lesion with a maximum diameter of 32 mm, containing partly amorphous and partly tooth-like radiopacities and a caudally displaced first permanent lower left molar (see Fig. 1). Furthermore, the follicle of the second lower left permanent molar was absent.

With the working diagnosis of a compound/complex odontoma, surgical treatment was planned. Under general anesthesia, the lesion was enucleated through a transoral approach, leaving the lower left first molar untouched. The residual surgical cavity was filled with a collagen sponge. The postoperative course was uneventful.

## Histology and Molecular Findings

**Fig. 2:**
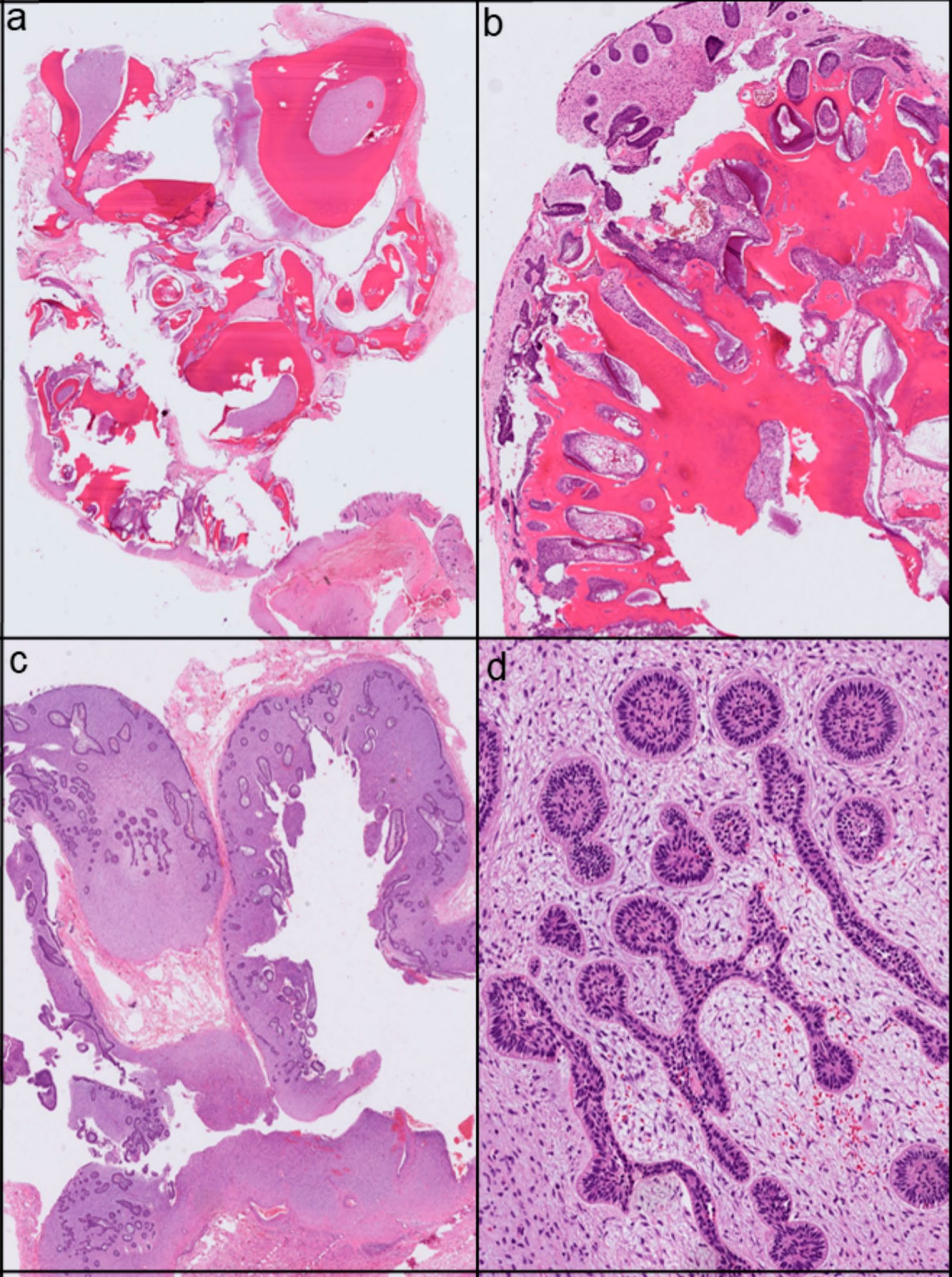
(**a**,** b**) Hard tissue components of the ameloblastic fibro-odontoma, illustrating the odontogenic structures. (**c**,** d**) Soft tissue components resembling ameloblastic fibroma, highlighting the stromal and epithelial elements

Pathological microscopic examination of the enucleation revealed a mixed epithelial and mesenchymal tumor consisting of a cell-rich fibrous stroma with some myxoid features and monomorphic spindle-shaped to stellate-like fibroblasts. Additionally, there are fields of epithelium with palisading of peripheral nuclei and subnuclear vacuoles and centrally a stellate reticulum consistent with ameloblastic morphology. In the other sections, more hard elements are observed with anastomosing fields of dentin embedded in the previously described stroma, and there are strips of the ameloblastic epithelium. See Fig. 2 for histological images.

Next-generation sequencing (NGS) was performed using the TSO500 panel and NextSeq sequencer (Illumina). Data was analyzed using Franklin (Genoox). NGS showed a likely pathogenic mutation in the *FGFR1* (NM_023110.3): c.1638 C > G (p.Asn546Lys), with a variant allele frequency of 14.9%. This *FGFR1* mutation is located in the kinase domain and has been described to result in the activation of *FGFR1* and increased transforming potential [5]. No other mutations were detected in this 523 gene panel. Whole transcriptome sequencing did not yield any results.

## Discussion

This case report describes an AFO with an *FGFR1* mutation. FGFRs (Fibroblast Growth Factor Receptors) are crucial in cellular functions like proliferation, differentiation, and survival by regulating pathways such as RAS-ERK/MAPK. When Fibroblast Growth Factor (FGF) binds to FGFR, it initiates a cascade activating key proteins—RAS, RAF (e.g., BRAF), MEK, and ERK—promoting cell growth and differentiation [6]. Mutations in *FGFR* and *BRAF* disrupt this pathway, contributing to tumorigenesis in various cancers [7].

The molecular pathogenesis of odontogenic tumors is not fully understood; however, the MAPK/ERK pathway is known to play a role in a subset of tumors with ameloblastic features, such as ameloblastic fibroma, AFO, and ameloblastoma [3, 4, 8]. Notably, *BRAF* V600E mutations are frequently identified in these tumors, and *FGFR* mutations have also been reported in ameloblastomas [7, 9, 10]. Therefore, it is unsurprising that, besides *BRAF*, an *FGFR* mutation has now been identified in AFO.

The classification of AFO has evolved over time. Previously, it was classified as a distinct entity among mixed odontogenic tumors [11]. However, in the 4th edition of the World Health Organization Classification of Head and Neck Tumours: Odontogenic and Maxillofacial Bone Tumors, published in 2017, AFO was redefined as part of the continuum of developing odontomas [1]. This perspective was reaffirmed in the 5th edition published in 2022, emphasizing that AFO is not a neoplastic entity but represents the early stages of odontoma development [12].

There may be a grey area in which certain tumors cannot be definitively classified as either complex odontomas or AFOs. When a tumor exhibits features of a complex odontoma but also contains focal components resembling dental papilla– morphologically similar to ameloblastic fibroma– differentiating between a complex odontoma and an AFO can be challenging. However, in cases where tumors exhibit pronounced ameloblastic fibroma-like features and distinct hard tooth-like formations (like complex odontoma), a diagnosis of AFO should probably be rendered. Some cases may fall within a spectrum between well-defined odontogenic entities. However, the presence of *BRAF* or *FGFR* mutations could suggest that the tumor aligns more closely with an AFO rather than a (developing) complex odontoma.

## Conclusion

The finding of an *FGFR1* mutation in AFO may suggest a biological relationship between odontogenic tumors with ameloblastic features, such as ameloblastoma, ameloblastic fibroma, and AFO. This mutation could indicate a neoplastic process rather than an odontoma. Further molecular studies are needed to explore whether AFO represents a distinct entity from ameloblastic fibroma and odontoma.

## Data Availability

No datasets were generated or analysed during the current study.
